# *Nod2* Deficiency in mice is Associated with Microbiota Variation Favouring the Expansion of mucosal CD4+ LAP+ Regulatory Cells

**DOI:** 10.1038/s41598-018-32583-z

**Published:** 2018-09-24

**Authors:** A. Butera, M. Di Paola, L. Pavarini, F. Strati, M. Pindo, M. Sanchez, D. Cavalieri, M. Boirivant, C. De Filippo

**Affiliations:** 1 Pharmacological Research and Experimental Therapy Section, National Center for Drug Research and Evaluation, Istituto Superiore di Sanità, Rome, Italy; 2 Department of Neuroscience, Psychology, Drug Research and Child Health (NEUROFARBA), University of Florence, Meyer Children Hospital, Florence, Italy; 3 Department of Biology, University of Florence, Sesto Fiorentino, Florence, Italy; 4 Research and Innovation Centre, Fondazione E. Mach, S. Michele all’Adige, Trento, Italy; 5 Institute for Research in Biomedicine (IRB), Università della Svizzera italiana, Bellinzona, Switzerland; 6 Cytometry Unit - Core Facilities, Istituto Superiore di Sanità, Rome, Italy; 7 Institute of Biology and Agrarian Biotechnology (IBBA), National Research Council (CNR), Pisa, Italy

## Abstract

Nucleotide-binding Oligomerization Domain-2 (*NOD2*) mutations are associated with an increased risk to develop Crohn’s Disease. In previous studies, we have shown that *Nod2−/−* mice manifest increased proportion of Lamina Propria (LP) CD4+ LAP+ Foxp3− regulatory cells, when compared with *Nod2*+/+ mice, while CD4+ Foxp3 + regulatory cells were not affected. Here, we investigated the *Nod2* gut microbiota, by 16S rRNA pyrosequencing, at steady state and after TNBS-colitis induction in mice reared separately or in cohousing, correlating the microbial profiles with LP regulatory T cells proportion and tissue cytokines content. We found that enrichment of *Rikenella* and *Alistipes* (*Rikenellaceae*) in *Nod2−/−* mice at 8 weeks of age reared separately was associated with increased proportion of CD4+ LAP+ Foxp3− cells and less severe TNBS-colitis. In co-housed mice the acquisition of *Rickenellaceae* by *Nod2*+/+ mice was associated with increased CD4+ LAP+ Foxp3− proportion and less severe colitis. Severe colitis was associated with enrichment of gram-negative pathobionts (*Escherichia* and *Enterococcus*), while less severe colitis with protective bacteria (*Barnesiella*, *Odoribacter* and *Clostridium IV*). Environmental factors acting on genetic background with different outcomes according to their impact on microbiota, predispose in different ways to inflammation. These results open a new scenario for therapeutic attempt to re-establish eubiosis in Inflammatory Bowel Disease patients with NOD2 polymorphisms.

## Introduction

Nucleotide-binding Oligomerization Domain 2 (NOD2) is an innate immune cytoplasmic microbial receptor, belonging to the nucleotide-binding oligomerization domain, leucine-rich repeat family (NLR). Several genetic studies have highly correlated mutations in *NOD2* domain with the development of Crohn’s disease (CD)^1–4^, though the mechanisms involved are still unclear, and perhaps linked to the NOD2 involvement in multiple processes, such as the preservation of intestinal barrier integrity and immune homeostasis, autophagy and balance of the gut microbiota composition^5–7^.

It has been reported that while normal NOD2 supports responses to gut commensals, and thus control organisms penetrating the intestinal lamina propria (LP), abnormal NOD2, with decreased function resulting from a polymorphism, exhibits a heightened inflammatory response due to blunted bacterial clearance^8,9^. However, *Nod2−/−* mice, despite showing increased LP CD4+ T cell-IFN-γ production, increased intestinal permeability and bacterial translocation^10,11^, do not show signs of overt intestinal inflammation. In previous studies, we have shown that *Nod2−/−* mice manifest increased colonic permeability, but this feature was associated with increased proportion of LP CD4+ LAP+ Foxp3− regulatory cells and increased TGF-β tissue content, when compared with *Nod2*+/+ mice, while CD4+ Foxp3+ regulatory cells were not affected. These features were associated with a less severe colitis induced by 2,4,6-Trinitrobenzenesulfonic acid (TNBS) in *Nod2−/−* mice when compared to *Nod2*+/+ mice^12–14^.

It has been hypothesized that *NOD2* mutations or deficiency can concur to shape the gut microbiota profiles, and thus the inflammatory disease development. Previous studies in humans showed gut microbiota alterations in mucosa of CD patients with *NOD2* homozygous mutations^15^. In mice model, several studies reported the key role of *Nod2* in the regulation of gut microbiota homeostasis^7,15–20^. Microbiota modifications related to *Nod2* deficiency were also found strictly dependent on animal housing conditions^21,22^.

In the present study, we investigated the microbial and mucosal immune profiles of *Nod2−/−* and *Nod2*+/+ mice reared independently or cohoused, at the steady state and after TNBS-colitis induction, to understand the impact of genetic background and environment on microbiota profiles and immune response.

## Results and Discussion

**Figure 1:**
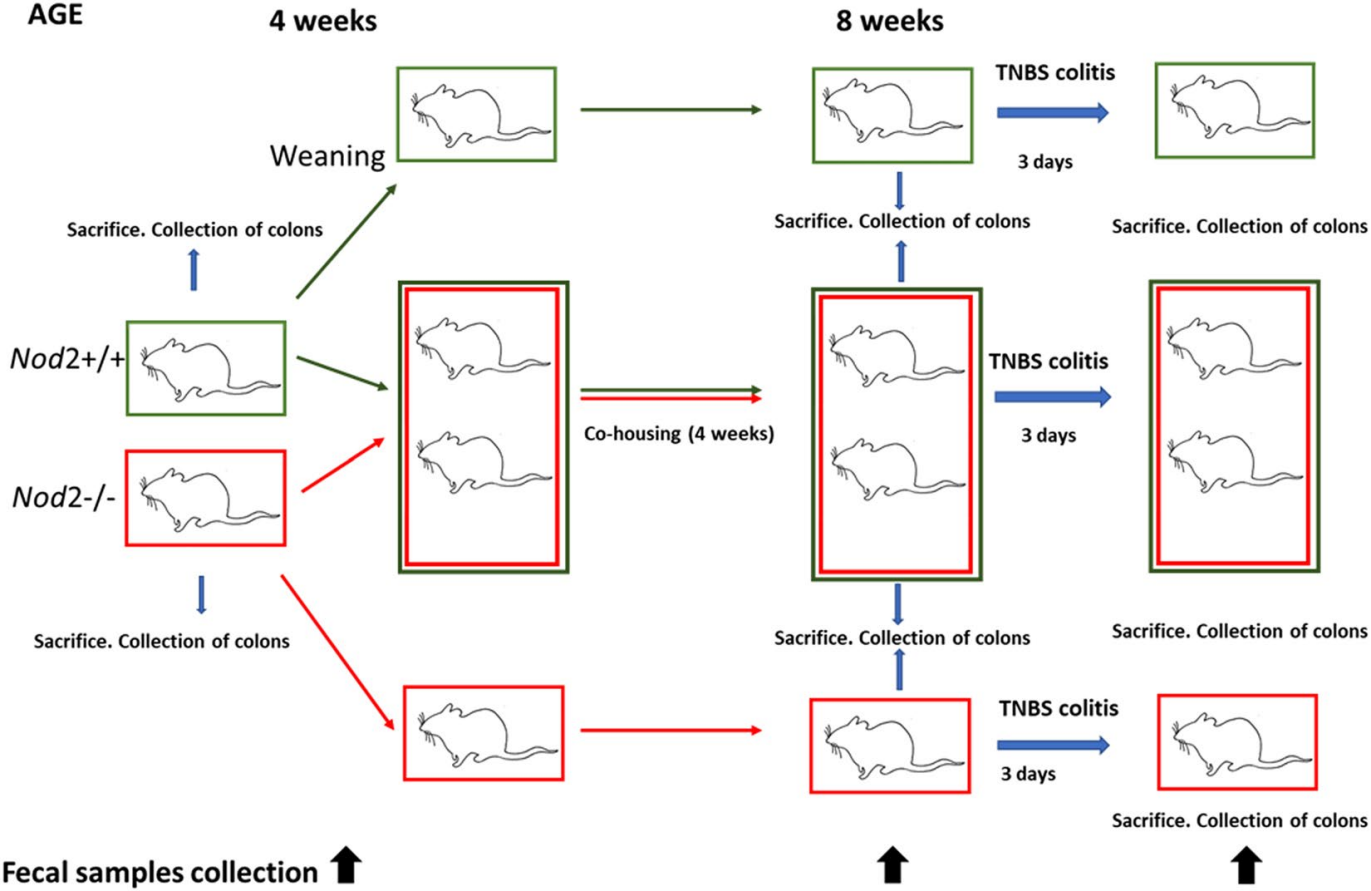
Experimental design on *Nod2*−/− and *Nod2*+/+ mice to investigate the effect of genetic background and housing conditions on microbiota-immune-system interactions. Black arrows indicate faecal sample collection for microbiota analysis at different time points: (i) at 4 weeks of age, (ii) at 8 weeks of age, and (iii) at day 3 after TNBS-colitis induction in cohoused and reared separately mice groups, respectively. At the three time points, mice were sacrificed to collect colon and test the proportion of LP CD4+ LAP+ Foxp3− T cells and tissue cytokines content.

In order to evaluate the effect of *Nod2* genotype on microbiota-immune-system interactions, we performed meta-taxonomic analysis of gut microbiota in *Nod2−/−* and *Nod2*+/+ mice at different time points and housing conditions (Fig. 1 for experimental design and Methods).

### Nod2−/− and Nod2+/+ mice show a clear separation of microbiota composition at 4 weeks of age

**Figure 2:**
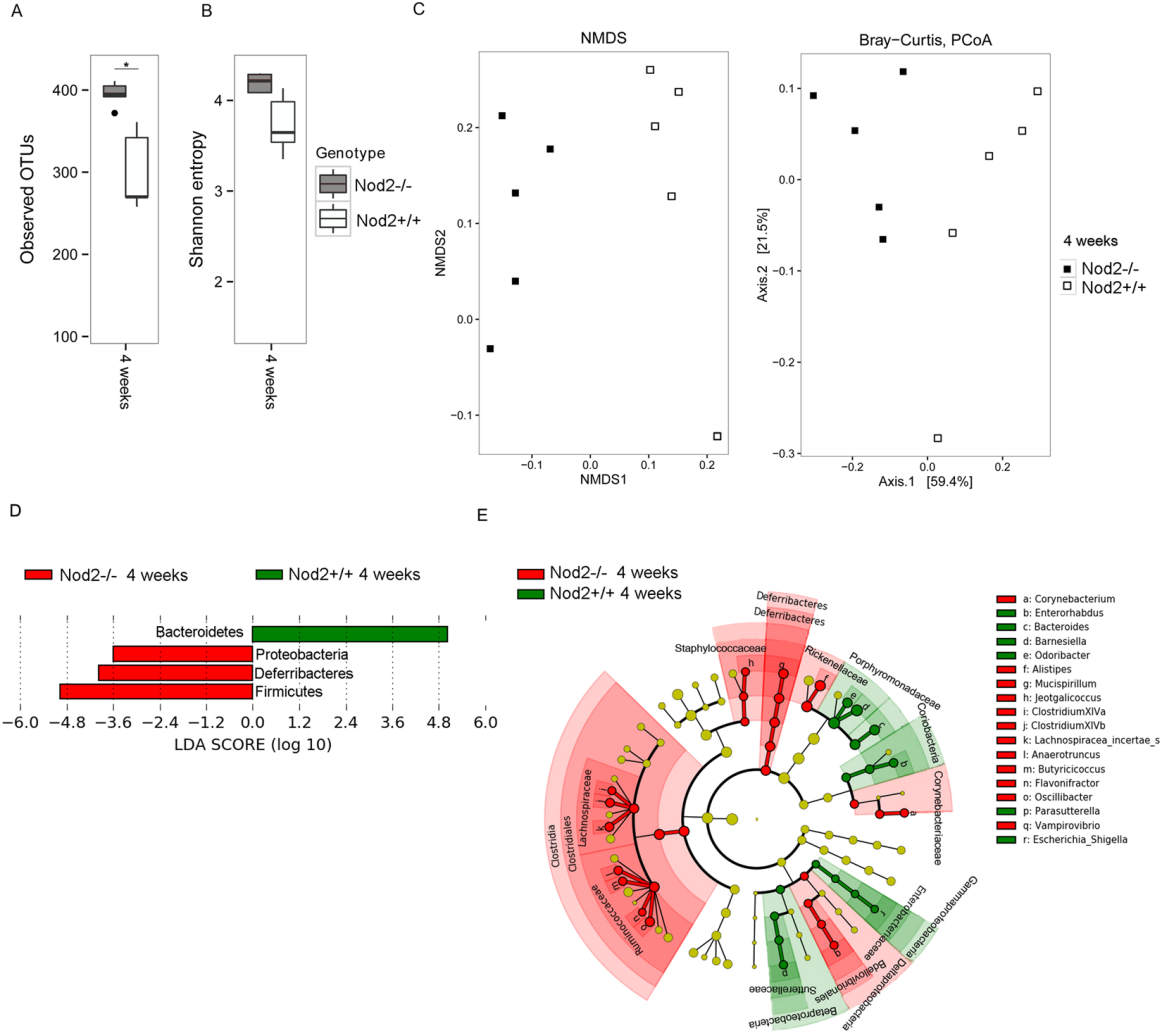
Microbiota comparison in mice by different genetic background. (**A**–**C**) Alpha and beta diversity measures at 4 weeks of age. (**A**,**B**) Alpha diversity. Box plots of (**A**) number of observed OTUs and (**B**) Shannon entropy in *Nod2*−/− and *Nod2*+/+ mice. Statistically significant differences were calculated by pairwise comparisons using Student t test with FDR correction; *p-value < 0.05. (**C**) Beta diversity. NMDS and Principal coordinate analysis (PCoA) based on the Bray-Curtis distance metric (PERMANOVA, p-value = 0.015). (**D**,**E**) LEfSe analysis shows a statistically significant enrichment based on different genotypes at 4 weeks of age. (**D**) Differences in bacterial phyla between *Nod2*−/− and *Nod2*+/+ mice. (**E**) Cladogram showing the most discriminative bacterial clades by comparison of *Nod2*−/− and *Nod2*+/+ microbiota. Coloured regions/branches indicate differences in the bacterial population structure between *Nod2*−/− and *Nod2*+/+ mice at 4 weeks. Regions in red indicate clades that were enriched in *Nod2*−/− mice, while regions in green indicate clades that were enriched in *Nod2*+/+ mice. LEfSe results indicate a sequentially significant ranking between the two groups (Alpha value = 0.05 for the factorial Kruskal-Wallis test among classes; the threshold for the logarithmic LDA score was 2.0).

We found differences immediately before weaning (4 weeks of age) in the gut microbiota composition between *Nod2−/−* and *Nod2*+/+ mice (Fig. 2). At this time, alpha diversity analysis (Fig. 2A, B; observed OTUs and Shannon entropy estimation), showed a higher microbial richness in *Nod2−/−* compared to *Nod2*+/+ mice (Observed OTUs p = 0.03 t test, FDR correction).

Beta diversity analysis, calculated by using Non-metric Multi-Dimensional Scaling (NMDS) and Principal Coordinates Analysis (PCoA) on Bray-Curtis distances, discovered a clear separation between the two mice groups according to the different genotypes, indicating a great variability of microbial profiles between *Nod2−/−* and *Nod2*+/+ mice (Fig. 2C).

By meta-taxonomic analysis, we found different abundances in Bacteroidetes and Firmicutes between *Nod2−/−* and *Nod2*+/+ mice (by Linear discriminant analysis Effect Size-LEfSe; Fig. 2D). In particular, we observed that, although the proportion between Bacteroidetes and Firmicutes was well-balanced in *Nod2−/−* mice, Firmicutes was enriched when compared to *Nod2*+/+ mice, while Bacteroidetes was enriched in *Nod2*+/+ compared to *Nod2−/−* mice (Bacteroidetes and Firmicutes: in *Nod2−/−* 48.9% and 46.6% and in *Nod2*+/+ 72.6% and 25.4%, respectively; Supplementary Tables S1–S2). Proteobacteria and Deferribacteres phyla were also enriched in *Nod2−/−* mice, but their abundances did not exceed the 1% (Fig. 2D).

These results are in agreement with previous observations showing in weaning *Nod2−/−* mice roughly twice the number of OTUs compared to *Nod2*+/+ mice and a clustering of microbiota composition between *Nod2*+/+ and *Nod2−/−* mice, with a significant abundance in Firmicutes in ileum content of adult and young *Nod2−/−* mice^15^. In addition, the increased abundance of Firmicutes observed in the present study in Nod2*−/−* mice is in agreement with initial observations reported in biopsies sampled from non-inflamed mucosal area of the terminal ileum of CD patients that were homozygous for the Leu1007fs (SNP13) compared to CD patients that were homozygous for the WT NOD2 allele^15^. Similar results were also obtained in a subsequent study involving CD macroscopically disease-unaffected regions of the ileum^23^.

Regarding the Firmicutes phylum, LEfSe analysis showed a statistically significant enrichment of *Lachnospiraceae* in *Nod2−/−* (19.7%) compared to *Nod2*+/+ mice (5.4%), and *Ruminococcaceae* (6.4% in *Nod2−/−* vs 2.0% in *Nod2*+/+ mice; Fig. 2E). Other minor representative families, such as *Corynebacteriaceae* (Actinobacteria), *Bdellovibrionaceae* (Proteobacteria) and *Deferribacteriaceae* (Deferribacteres) were found enriched in *Nod2−/−* with respect to *Nod2*+/+ mice (Fig. 2D–E; Supplementary Table S2).

At lowest taxonomic level (Fig. 2E), in *Nod2−/−* mice we found enrichment of several genera, such as *Alistipes* (Bacteroidetes; 8.8% vs 2.5% in *Nod2−/−* compared to *Nod2*+/+ mice), *Mucispirillum* (Deferribacteres; 2.3% vs 0.3% in *Nod2−/−* compared to *Nod2*+/+ mice) and other minor representative genera belonging to Firmicutes (*Anaerotruncus*, *Butyrricicoccus*, *Clostridium cluster XIVa* and *XIVb*, *Flavonifractor*, *Jeotgalicoccus*, *Lachnospiracea incerte sedis*, and *Oscillibacter)* together with *Corynebacterium* (Actinobacteria) (Supplementary Table S2).

In *Nod2*+/+ mice, we observed a statistically significant enrichment in *Porphyromonadaceae* (Bacteroidetes; 50.62% vs 34.6% in *Nod2*+/+ compared to *Nod2−/−* mice), *Enterobacteriaceae*, *Sutterellaceae* (Proteobacteria), and *Coriobacteriaceae* (Actinobacteria) families (Fig. 2E; Supplementary Table S2). *Barnesiella*, a member of the *Porphyromonadaceae* family, known to regulate the microbial composition^24^ and inhibit clostridia and other potentially harmful microbes^25^, was the most abundant genus (28.3% vs 16.7% in *Nod2*+/+ compared to *Nod2−/−* mice). We found also enrichment of *Odoribacter* (*Porphyromonadaceae*; 2.1% vs 1% in *Nod2*+/+ compared to *Nod2−/−* mice), *Bacteroides* (Bacteroidetes; 4% vs 0.7% in *Nod2*+/+ compared to *Nod2−/−* mice), and other minor representative genera, including the pathobionts *Escherichia/Shigella* (*Enterobacteriaceae*), *Enterorhabdus* (*Coriobacteriaceae*)^26^ and *Parasutterella* (*Sutterellaceae*)^27^ (Fig. 2E; Supplementary Table S2).

These results are partially in agreement with previous reported data^28^ that showed an over-representation of *Alistipes*, in *Nod2−/−* mice microbiota and a depletion in bacteria belonging to Proteobacteria, such as *Escherichia/Shigella* genus^20^, that in our study was enriched in *Nod2*+/+ mice.

To predict how taxonomic differences between microbiota of *Nod2−/−* and *Nod2*+/+ mice could affect their microbial metabolic potential, we applied PICRUSt (Phylogenetic Investigation of Communities by Reconstruction of Unobserved States)^29^ (see Methods). LEfSe analysis, performed on PICRUSt output (Supplementary Fig. S1), showed differentially enriched functional classes (KEGG categories) between *Nod2−/−* and *Nod2*+/+ mice. Interestingly, although in both microbiomes we identified a common core of metabolic capabilities related to carbohydrate, lipid and amino acid metabolism, in *Nod2*+/+ mice we observed significant enrichment of functions related to lipopolysaccharides (LPS) biosynthesis. It is well known that LPS is a component of cell wall of Gram-negative bacteria. This result correlates with the observed higher abundance of Gram-negative taxa, such as Bacteroidetes phylum, *Porphyromonadaceae* family, *Escherichia/Shigella* and *Parasutterella* genera in *Nod2*+/+ mice (Fig. 2D–E) compared to the abundance of Firmicutes (Gram-positive) observed in *Nod2−/−* mice.

In *Nod2−/−* mice, we found an enrichment of functions related to cell motility (flagellar assembly, bacterial chemotaxis and bacterial motility proteins), membrane transport (secretion system, sulfur relay system, ansamycin biosynthesis), and cellular processes and signaling. These functions could be associated to the potential ability of some microorganisms to adapt and respond to external stimuli.

### Colonic tissue Immunologic profile

In order to correlate microbiota composition with mucosal immune-profile, we analyzed *Nod2*+/+ and *Nod2−/−* colonic tissue samples from 5 mice/group for cytokine mRNA tissue content and proportion of LP CD4+ LAP+ Foxp3− regulatory cells. We found a significant increase in the percentage of LP CD4+ LAP+ Foxp3− regulatory cells in *Nod2−/−* mice when compared with *Nod2*+/+ mice (6.28 ± 1.8 vs 2.5 ± 1.4, mean ± SE, p < 0.05, respectively). As regard colonic tissue cytokine mRNA content, in *Nod2−/−* mice we observed a significant increase of TGF-β mRNA content compared to *Nod2*+/+ mice (8.3 ± 2 vs 2 ± 0.4 AU, mean ± SE, p < 0.05, respectively) and an increase (although not significant) in IL-10 mRNA content (7.2 ± 2.2 vs 3.6 ± 0.1 AU, mean ± SE, p = 0.31, respectively).

Notably, as described above, the microbiota of *Nod2−/−* mice at 4 weeks of age was enriched of *Lachnospiraceae* and *Ruminococcaceae*, two bacterial families encompassing known butyrate-producers^30^, and other protective bacteria, such as *Clostridium cluster XIVa*, involved in CD4+ Foxp3+ regulatory T cells accumulation^31,32^, *Alistipes* and *Butyriciccous*, two genera reported to be depleted in human IBD samples^28,33^ and able to protect from colitis in mice^33,34^ (Fig. 2E). On the other hand, *Nod2*+/+ microbiota was relatively enriched of Gram-negative and LPS-producer pathobionts, such as *Escherichia/Shigella*, and *Parasutterella* (Fig. 2E), found in association with inflammation, colitis and enriched in the gut microbiota of CD patients^27,35,36^.

Taken together our data show, at this time point, an association between the enrichment of protective bacteria in *Nod2−/−* microbiota and the increase of LP CD4+ LAP+ Foxp3− regulatory cells and anti-inflammatory cytokines.

### Change of cage, grouping with other mice from different mothers minimize the difference in microbiota observed before weaning in mice reared separately

At weaning (4 weeks of age), some male mice from different mothers/cages were grouped in new cages and kept separated according to the genetic background (*Nod2−/−* or *Nod2*+/+).

**Figure 3:**
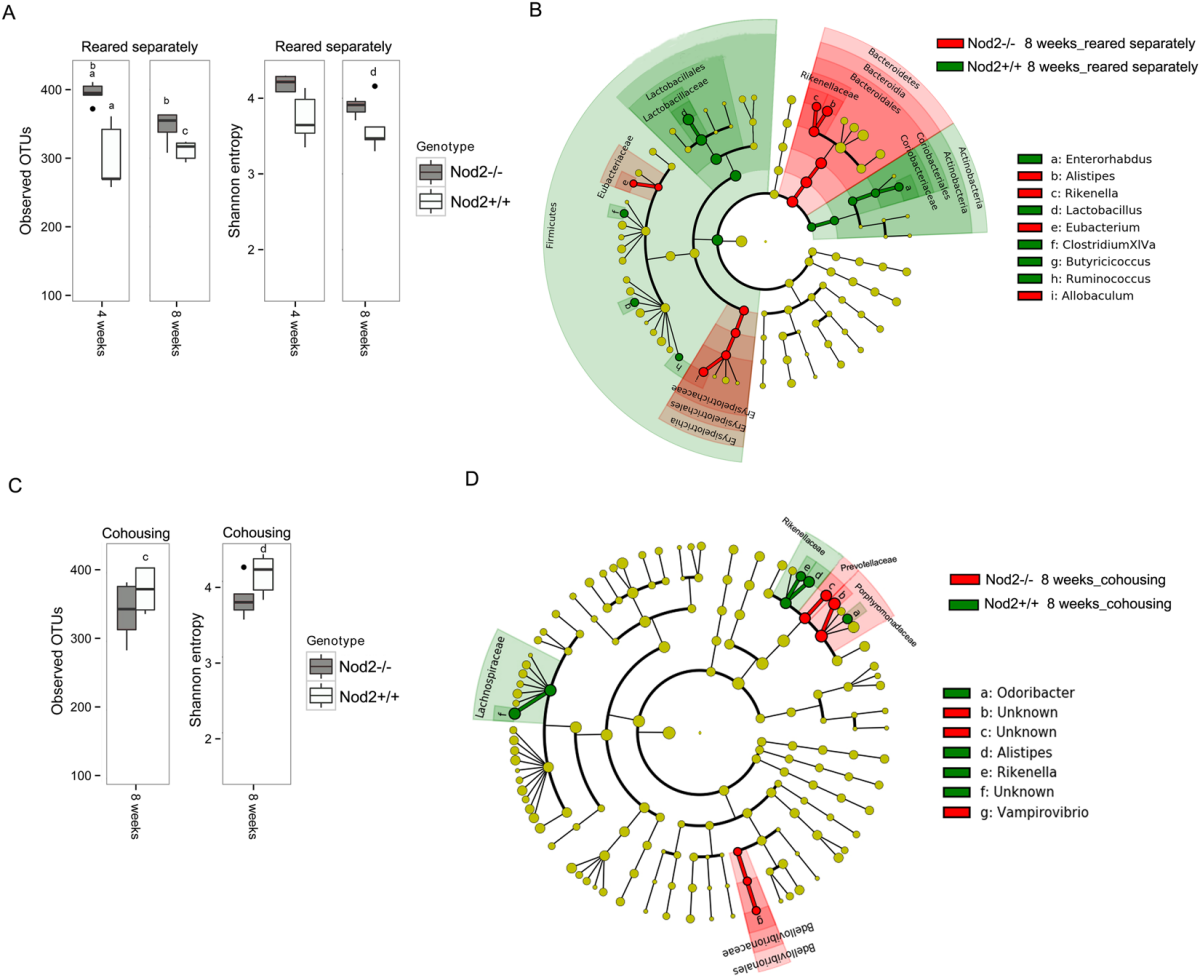
Microbiota comparison between *Nod2*−/− and *Nod2*+/+ mice at 8 weeks of age in different housing conditions. (**A**,**C**) Alpha diversity measures in mice (**A**) reared separately and (**C**) in cohousing. Box plots of number of observed OTUs and Shannon entropy in *Nod2*−/− and *Nod2*+/+ mice (**A**) reared separately at 4 and 8 weeks of age and (**C**) in cohousing at 8 weeks of age. Statistically significant differences were performed by pairwise comparisons using Student t test with FDR correction. For each significant pairwise comparison p-value < 0.05 are indicated by different letters (“a”, “b”, “c”, and “d”). (**B**–**D**) Microbiota profile comparison in *Nod2*−/− and *Nod2*+/+ mice at 8 weeks (**B**) reared separately and (**D**) in cohousing. Cladogram showed the most discriminative bacterial clades identified by LEfSe analysis. Coloured regions/branches indicate differences in the bacterial population structure between *Nod2*−/− and *Nod2*+/+ mice at 8 weeks. Regions in red indicate clades that were enriched in Nod2−/− mice, while regions in green indicate clades that were enriched in *Nod2*+/+ mice. LEfSe results indicate a sequentially significant ranking between the two groups (Alpha value = 0.05 for the factorial Kruskal-Wallis test among classes; the threshold for the logarithmic LDA score was 2.0).

Analysis of faecal microbiota composition of mice reared separately at 8 weeks of age revealed a decrease in alpha diversity in *Nod2−/−* mice compared with microbiota of the same mice at 4 weeks (Observed OTUs p = 0.033 t test FDR correction; Fig. 3A,B).

Few differences in microbial profiles were observed considering the different genetic backgrounds, as indicated also by beta diversity measures (Fig. 3 and Supplementary Fig. S2).

With respect to the 4 weeks of age, at this time point (8 weeks) we observed that the proportion of Bacteroidetes and Firmicutes in the two groups of mice was unchanged (Supplementary Table S1). In particular, we found an increase of Bacteroidetes and a reduction of Firmicutes in *Nod2−/−* when compared with *Nod2*+/+ mice (Bacteroidetes 71% vs 57%; Firmicutes 27% vs 40%, respectively). The most abundant taxa, such as *Porphyromonadaceae* (54.8% vs 49.7% in *Nod2−/−* and *Nod2*+/+ mice respectively) and *Lachnospiraceae* families (11.9% vs 12.8% in *Nod2−/−* and *Nod2*+/+ mice respectively), and *Barnesiella* genus (23.8% vs 20.9% in *Nod2−/−* and *Nod2*+/+ mice respectively) were comparable in terms of relative abundance either in *Nod2−/−* or *Nod2*+/+ mice when reared separately (Supplementary Table S2). These data are partly in agreement with previously reported data that showed in colon of 10 weeks old *Nod2−/−*mice a higher proportion of Bacteroidetes and a lower proportion of Firmicutes^18^. However, although we observed small differences in the same direction, we did not observe a significant increase in bacteria assigned to the *Porphyromonadaceae* family and a concomitant significant decrease in bacteria from *Lachnospiraceae* in *Nod2−/−* mice, as reported in the above mentioned study. These differences might be due to the different samples evaluated (colons in the reported study, and faeces in the present study). However, by LEfSe analysis, some differential abundances in bacterial taxa between *Nod2−/−* and *Nod2*+/+ mice reared separately were still present at 8 weeks of age (Fig. 3B). In particular, in *Nod2−/−* mice, the enrichment of *Alistipes* (5.4%; *Rikenellaceae*) is still observable. In addition, at this time-point enrichment in *Eubacteriaceae* and *Eryspelotrichaceae* families, and genera <1% in relative abundance, such as *Rikenella* (*Rikenellaceae*), *Eubacterium* (*Eubacteriaceae*) and *Allobaculum* (*Eryspelotrichaceae*) are observed in *Nod2−/−* mice microbiota, confirming in part previously reported data^19^. In *Nod2*+/+ mice, *Lactobacillus* (24% vs 10% in *Nod2*+/+ and *Nod2−/−* mice respectively) was the predominant genus. *Coriobacteriaceae* family and genera minor than 1% in terms of relative abundance, such as *Butyrricicoccus*, *Clostridium XIVa*, *Enterohabdus* and *Ruminococcus* significantly differentiates the microbiota composition (Fig. 3B; Supplementary Table S2). These data suggest that in *Nod2−/−* and *Nod2*+/+ at 4 weeks of age (weaning) other factors such as maternal effect and/or cage effect, may magnify differences in the microbiota, which are minimized when a more heterogenic microbiota resulting from cohousing with mice of different mothers and from different cages is acquired. It is interesting to note that some genera as *Clostridium XIVa* and *Butyrricicoccus* that were enriched in *Nod2−/−* mice microbiota at 4 weeks of age are now enriched in the *Nod2*+/+ mice microbiota. At the same time, some family/genus enrichment (i.e. *Rikenellaceae*/*Alistipes*) persists in *Nod2−/−* mice also at this time-point. The persistence of a specific component of the microbiota might indeed be the expression of the influence of genetic differences (i.e. the absence of functional *Nod2* gene) between the two lineages. This view is reinforced by the recent demonstration that NOD2 deficiency has indeed a dominant effect on microbial dysbiosis^18,19^.

### Colonic tissue Immunologic profile in 8 weeks-old mice reared separately

To evaluate at this time point the proportion of LP CD4+ LAP+ Foxp3− regulatory cells and cytokine mRNA tissue content between *Nod*2*−/−* and *Nod*2+/+ mice reared separately, some mice (5 mice/group) were sacrificed at 8 weeks of age. As shown in Table 1, we observed differences in IL-10 and in TGF-β tissue content that were significantly higher in 8 weeks-old *Nod2−/−* mice than in *Nod2*+/+ mice when reared separately. As previously shown^12^, proportion of colonic CD4+ LAP+ Foxp3− regulatory cells were also significantly higher in *Nod2−/−* than in *Nod2*+/+ tissue samples. This result could be associated with high microbial richness, as observed by alpha diversity measure (Fig. 3A), and with the presence of protective bacteria, such as *Alistipes*, *Eubacterium*, *Allobaculum*, known for beneficial effects on the colon mucus barrier^37^, as observed by LEfSe analysis (Fig. 3B).

**Table 1:** Immune profile of mice at 8 weeks of age.

Mice	LAP+ Foxp3− cells (percentage of LP CD3+ CD4+ gated cells) mean ± SE	IL-10 (mRNA content in colonic tissue; Arbitrary Units) mean ± SE	TGF-β (mRNA content in colonic tissue; Arbitrary Units) mean ± SE
*Nod2*−/−	4.6 ± 1.0*	7 ± 1.2*	4.9 ± 0.9*
*Nod2*+/+	2.7 ± 0.3	4.1 ± 0.5	2.5 ± 0.2
*Nod2*−/− cohoused	2.6 ± 0.8**	4.3 ± 0.7	1.4 ± 0.1
*Nod2*+/+ cohoused	6 ± 1.1	6.7 ± 1.5	2.2 ± 0.6

Statistical significance by Mann–Whitney U-test: *p < 0.05 *Nod2*−/− vs *Nod2*+/+; **p < 0.05 *Nod2*−/− cohoused vs *Nod2*+/+ cohoused.

### Effect of co-housing on microbiota composition

In order to evaluate the microbiota components influencing the regulatory T cells and cytokines profile in mucosal tissue, we analysed microbiota composition in *Nod2−/−* and *Nod2*+/+ mice after 4 weeks of cohousing. When mice of the two different lineages were cohoused, an increase of alpha diversity in microbiota of *Nod2*+/+ mice was observed with respect to either the same mice at 4 weeks of age or mice of the same genetic background reared separately (Fig. 3A–C; p value < 0.05 *Nod2*+/+ cohousing vs reared separately). The differences in alpha diversity between cohoused *Nod2−/−* and *Nod2*+/+ were reduced when compared with mice reared separately (Fig. 3A–C), indicating similar species richness in gut microbial community and suggesting a possible sharing of microbial taxa. Indeed, few differences were observed between the two genetic backgrounds, as indicated by beta diversity (Supplementary Fig. S2). In particular, both *Nod2−/−* and *Nod2*+/+ mice showed similar abundances of *Lactobacillus* (14.83% vs 14.86% in *Nod2−/−* and *Nod2*+/+ mice respectively), *Barnesiella* (16.4% vs 15.75% in Nod2*−/−* and Nod2+/+ mice respectively), *Bacteroides* (1.74% vs 1.50% in *Nod2−/−* and *Nod2*+/+ mice respectively), as previously reported by Robertson *et al*.^21^, and *Allobaculum* (1.82% vs 1.06% in *Nod2−/−* and *Nod2*+/+ mice respectively) (Supplementary Table S2). However, LEfSe analysis showed very few differences, in particular related to *Porphyromonadaceae* family in *Nod2−/−* mice, that was previously (4 weeks of age) enriched in *Nod2*+/+ mice (Fig. 3D; Supplementary Table S2). Notably, in *Nod2*+/+ mice, an unknown genus belonging to *Lachnospiraceae* family was significantly enriched, together with the minor representative genera, such as *Alistipes* (6.44% vs 4.29% in *Nod2*+/+ and *Nod2−/−* mice respectively), *Rikenella* (0.85% vs 0.42% in *Nod2*+/+ and *Nod2−/−* mice respectively) that was previously (4 weeks of age) enriched in *Nod2−/−* mice (Fig. 3D; Supplementary Table S2).

In accordance with the above described data prediction of functional contribution of microbial communities by LEfSe analysis performed on PICRUSt results (Supplementary Fig. S3) showed that among the metabolic functions, an enrichment in functions related to membrane and intracellular structural molecules, in particular LPS biosynthesis, are now present in *Nod2−/−* cohoused mice, as opposite as the observed enrichment at 4 weeks of age in *Nod2*+/+ mice. This seems almost certainly due to the observed inverted proportion of Bacteroidetes between 4 and 8 weeks of age (Supplementary Table S1), and other Gram-negative bacteria (*Vampirovibrio*). The LPS biosynthesis is relevant in determining the homeostatic response to microbiota, therefore imbalance in its synthesis may influence the severity of colitis^38^.

In *Nod2*+/+ cohoused mice, enriched functions related to cell motility (Flagellar assembly, Bacterial chemotaxis and Bacterial motility proteins), membrane transport (sulfur relay system, ABC transporters) and ansamycin biosynthesis were found, as previously observed in *Nod2−/−* mice at 4 weeks of age.

Taken together the data leads to the conclusion that cohousing favours the sharing of microbiota components. These data are partially in contrast with previous data, which demonstrated no difference in microbiota composition in co-housed mice^21,22^. The duration of cohousing, short in our studies, mice housed together for the entire studies in the above cited studies^21,22^, and or the different method utilized in the present study for microbiota composition assessment and analysis may explain the different results.

### Colonic tissue Immunologic profile of cohoused mice

As for *Nod2−/−* and *Nod2*+/+ reared separately, 5 mice/group were sacrificed after 4 weeks of cohousing (8 weeks of age) for the assessment of colonic cytokine content and proportion of LP T regulatory cells. As shown in Table 1, in cohoused mice percentage of LP CD4+ LAP+ Foxp3− cells is higher in *Nod2*+/+ mice than in *Nod2−/−* mice. This latter showed values comparable to that observed in *Nod2*+/+ mice reared separately. This result could be associated with enrichment of *Alistipes* and *Rikenella* (both belonging to *Rikenellaceae* family) as observed by LEfSe analysis (Fig. 3B–D). In fact, the increased proportion of CD4+ LAP+ Foxp3− cells seems associated with enrichment in *Rickenellaceae* found in no-cohoused Nod2*−/−* mice microbiota and in co-housed *Nod2*+/+ mice microbiota. An increased abundance of *Alistipes* was recently found to be associated with administration of curcumin in mice^39^, a spice known for its beneficial effect on experimental colitis^40^ and possibly in patients with IBD^41^. Furthermore, it has been shown *in vitro* and in pre-clinical models, that curcumin is able to modulate bowel inflammation, through its ability to enhance IL-10-mediated effects^42^. Since we have previously shown that CD4+ LAP+ Foxp3− cells expansion is IL-10 dependent^43^ it is tempting to speculate that the variation in the proportion of CD4+ LAP+ Foxp3− cells observed in the different housing conditions might be related to the different enrichment in *Rickenellaceae*, particularly *Alistipes*. This hypothesis is reinforced by the observation made in the present study concerning the significant increase in IL-10 tissue content in non-cohoused *Nod2−/−* vs *Nod2*+/+ mice and the increased IL-10 tissue content in co-housed *Nod2*+/+ vs *Nod2−/−* mice. Further observations suggest a link between *Alistipes* and CD4+ LAP+ Foxp3− cells. It has been reported, in a study utilizing faecal microbiota transplantation from colonic cancer patients and healthy individuals into germ-free mice that, among others, *Alistipes* positively correlated with increased tumour burden in recipient mice^13^. It has also been reported, in the context of the suggestion that T regulatory cells promote tumour development and metastasis by inhibiting the proliferation of effector T lymphocytes, an increased percentage of LAP+ CD4+ T cells in peripheral blood and in tumour tissue of patients with colorectal cancer that correlates with tumour progression via the CD4+ LAP+ Foxp3− production of IL-10 and TGF-β^44^. Further studies, directly investigating the effect of *Alistipes* on the generation of CD4+ LAP+ Foxp3− regulatory cells are needed to confirm this hypothesis. Since a recent study^25^, aimed at understanding the interaction and changes of bacterial communities (e.g. microbial-interaction network and dynamic model between *Barnesiella* and *Clostridium difficile*), confirms once more, that microbial networks, rather than single strains or species, are associated to complex immune phenotypes, it is also likely that the unclassified microbial genus belonging to *Lachnospiraceae* that showed the same trend of *Alistipes*, and others beneficial bacteria (i.e. *Rickenella*, *Eubacterium* and *Odoribacter*), could create a multi-genera network associated to the generation of CD4+ LAP+ Foxp3− cells.

As additional confirmatory finding, colonic TGF-β mRNA tissue content was higher in *Nod2−/−* than in *Nod2*+/+ mice reared separately (Table 1), as observed in our previous study^12^, but was higher in *Nod2*+/+ mice than in *Nod2−/−* mice when the two groups of mice were co-housed (Table 1). Taken together, our data suggest that *Nod2* deficiency and related differences in microbiota composition impact on immune-response, especially on the proportion of LAP+ CD4+ T regulatory cells. This observation is not in agreement with recent studies using *Nod2* deficient mice transplanted with wild-type bone marrow cells and *vice versa*^17^. These studies showed that epithelial barrier functions and GALT composition are related to the lack of *Nod2* in the hematopoietic cells and that the dysbiosis observed in *Nod2−/−* mice in these studies, does not play a crucial role in the control of immune-response at the homeostasis. In the present study, indeed we show that the observed changes in mucosal immune-profile are clearly related to the acquired microbial profiles as demonstrated by the observations reported in co-housed mice. These discrepancies could be partially reconciled by the observation that in the above mentioned studies regulatory cells and cytokines were not analysed.

### TNBS-colitis shows different and opposite severity in Nod2*−/−* and Nod2+/+ mice reared separately or cohoused

**Figure 4:**
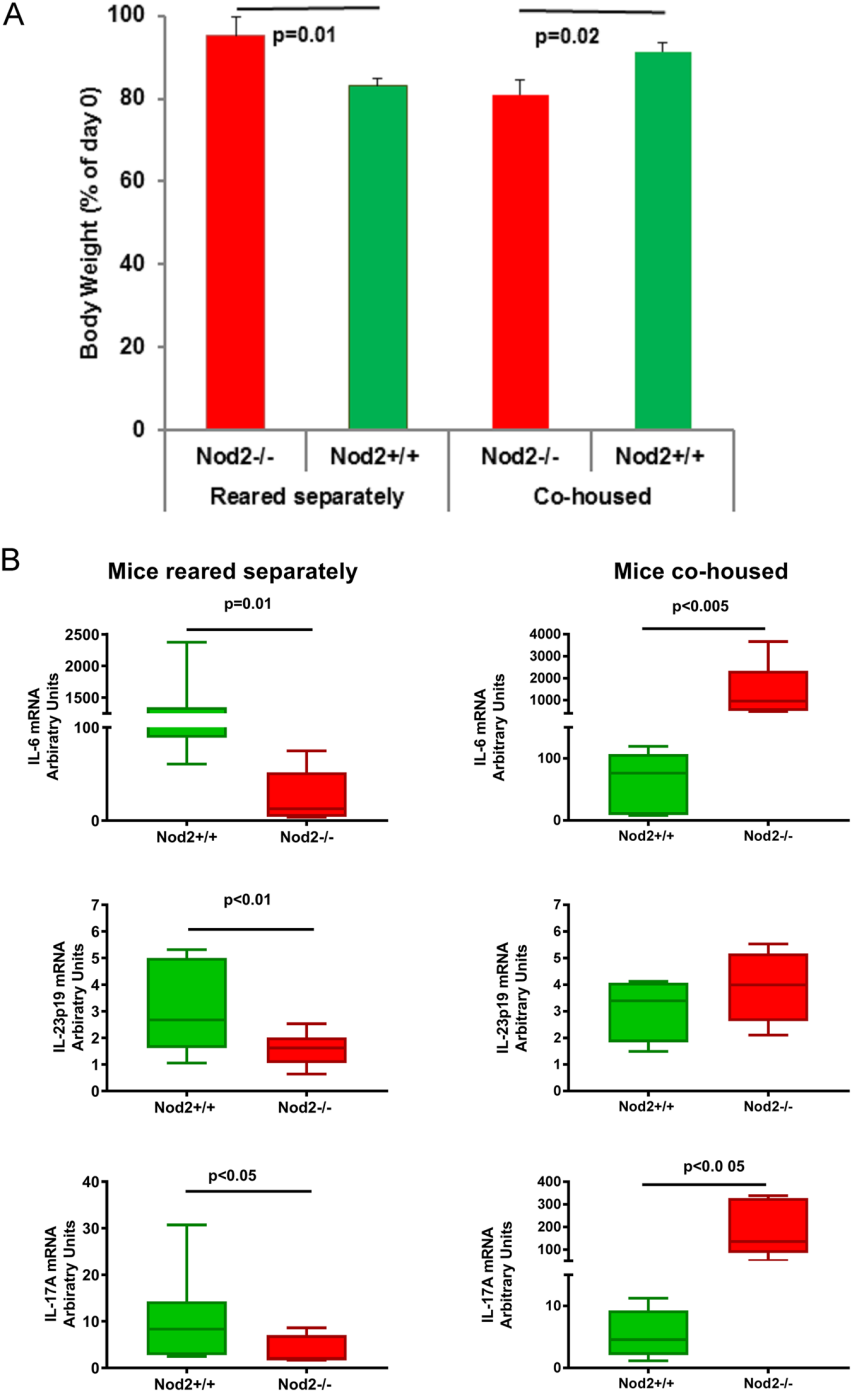
*Nod2*−/− mice exhibit less severe TNBS-colitis compared with *Nod2*+/+ mice reared separately, but more severe colitis than *Nod2*+/+ mice when co-housed. Mice were administered intra-rectal TNBS to induce TNBS-colitis (see methods). **(A)** Body weight changes of mice. Each point represents mean ± SE weight at day 3 from induction of colitis (day 0) expressed as percentage of day 0 weight and derived from 5 mice/group; p = 0.01 and p = 0.02 (Student’s t test) *Nod2*−/− vs *Nod2*+/+ mice reared separately or co-housed, respectively. (**B**) Cytokine mRNA colonic tissue content. Mice were administered intra-rectal TNBS to induce TNBS-colitis. At day 3 from colitis induction mice were sacrificed and colons were removed to be processed as described in material and method section. Statistical differences were evaluated by Mann–Whitney U-test.

In order to confirm previously reported data on different severity of TNBS colitis in *Nod2−/−* and *Nod2*+/+ mice and evaluate the relationship with microbiota profiles, in each of the four groups of mice at the age of 8 weeks, colitis was induced by intra-rectal administration of TNBS. As previously reported^12^, when reared separately, *Nod*2*−/−* mice showed a significant less severe colitis compared to *Nod2*+/+ mice; as opposite, in cohoused groups, *Nod2−/−* mice showed a more severe colitis when compared to *Nod2*+/+ mice (Fig. 4). Additionally, *Nod2*+/+ mice co-housed showed a less severe colitis when compared to TNBS colitis induced in *Nod2*+/+ mice reared separately. Indeed the course of colitis was comparable in both *Nod2*+/+ cohoused and *Nod2−/−* mice reared separately, as indicated by the weight loss (Fig. 4A). The different severity of colitis was also reflected by the colonic mRNA content of proinflammatory cytokines (Fig. 4B). We have previously demonstrated by adoptive transfer studies^12^ that the observed protection from TNBS-colitis in *Nod2−/−* mice reared separately was dependent on the increased proportion of LP CD4+ LAP+ Foxp3− cells observed in these mice. In the present study, severity of colitis was strictly related to the different proportion of CD4+ LAP+ Foxp3− observed before TNBS treatment (at 8 weeks of age; Table 1) in *Nod2−/−* and *Nod2*+/+ mice under different housing conditions, confirming the direct influence of CD4+ LAP+ Foxp3− regulatory cells on severity of colitis. Although the observation of less severe TNBS colitis in *Nod2−/−* mice reared separately from *Nod2*+/+ is in contrast with previous studies in which an increased severity of colitis in *Nod2−/−* mice compared with *Nod2*+/+ mice was reported^10^, it is possible that the observed differences reside in differences related to the various facilities favouring or not a pro-inflammatory microbiota in *Nod2−/−* mice.

**Figure 5:**
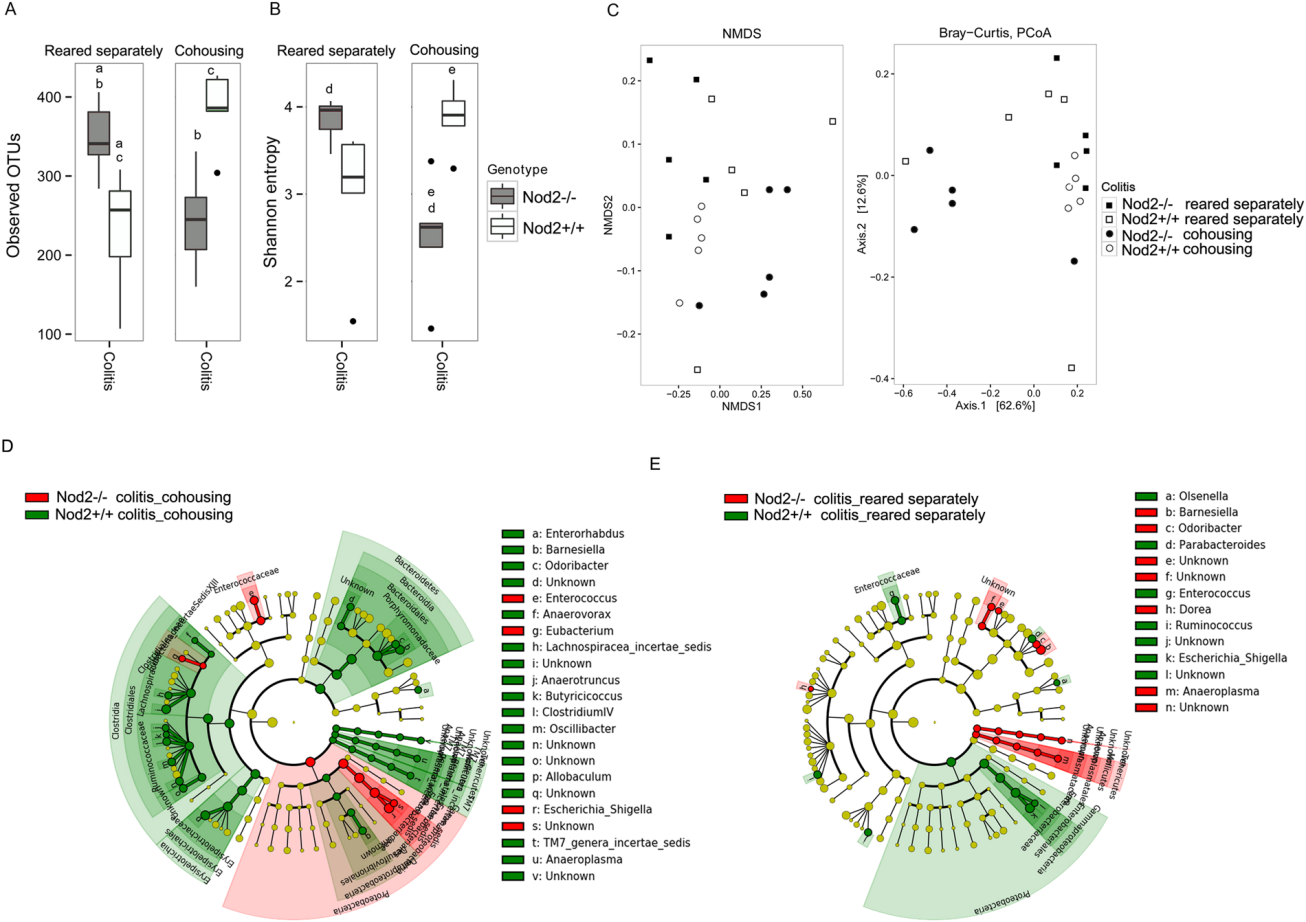
Microbiota comparison in *Nod2*−/− and *Nod2*+/+ mice after TNBS-induced colitis. (**A**–**C**) Alpha and beta diversity measures. (**A**,**B**) Alpha diversity. Box plots of (**A**) number of observed OTUs and (**B**) Shannon entropy in *Nod2*−/− and *Nod2*+/+ mice cohoused or reared separately after 3 days of TNBS-induced colitis. Pairwise comparisons using t test with FDR correction. For each significant pairwise comparison p-value < 0.05 are indicated by different letters (“a”, “b”, “c”, and “d”, “e”) (**C**) Beta diversity. NMDS and PCoA obtained by Bray-Curtis distances (PERMANOVA, p = 0.001). (D-E) LEfSe analysis. Cladogram showed the most discriminative bacterial clades. Coloured regions/branches indicate differences in the bacterial population structure between *Nod2*−/− and *Nod2*+/+ mice after TNBS-induced colitis (**D**) in cohousing and (**E**) reared separately. LEfSe results indicate a sequentially significant ranking between the two groups (Alpha value = 0.05 for the factorial Kruskal-Wallis test among classes; the threshold for the logarithmic LDA score was 2.0).

**Figure 6:**
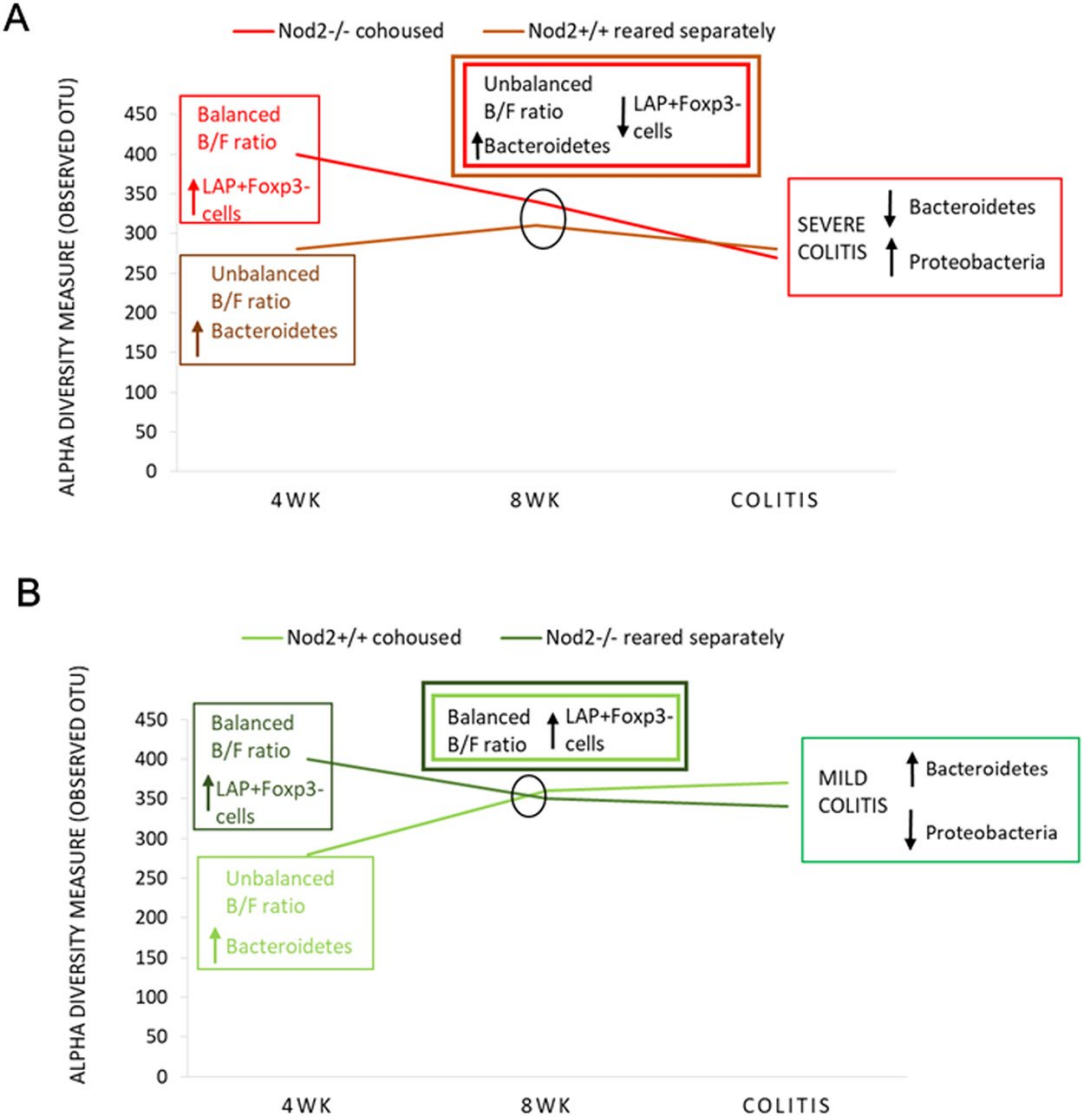
Summary of alpha diversity measure, microbial and immunologic profiles in *Nod2*−/− and *Nod2*+/+ mice reared separately or cohoused at different time points. Alpha diversity, Bacteroidetes/Firmicutes (B/F) ratio and CD4+ LAP+ Foxp3− levels were compared at 4 weeks, 8 weeks of age, and after TNBS-colitis. Interestingly, (**A**) in mice groups developing severe colitis (*Nod2*−/− cohoused and *Nod2*+/+ reared separately), progressive reduction of alpha diversity, unbalanced B/F ratio and reduction of CD4+ LAP+ Foxp3− proportion (at 8 weeks), and expansion of Proteobacteria (after TNBS-colitis) were observed, conversely to (**B**) *Nod2*−/− reared separately and *Nod2*+/+ cohoused developing less severe colitis.

As expected, severe colitis (as observed in *Nod2*+/+ mice reared separately, and in *Nod2−/−* mice co-housed) was associated with a reduction of alpha-diversity compared with the genotype and housing condition counterparts (Figs 5A, B and 6). This is in agreement with the notion reported in several human IBD studies that microbial richness is reduced in microbiota of patients affected by inflammatory diseases, especially IBD^45^. In addition, beta diversity (Fig. 5C) showed a clustering of cohoused *Nod2−/−* mice samples (more severe colitis) from samples of *Nod2*+/+ cohoused mice and *Nod2−/−* mice reared separately (both developing less severe colitis).

Due to the mice coprophagy, co-housing between two different genetic lineages represents a mean to evaluate the effects of acquisition of transferable microbiota compounds from one lineage to the other. The first striking effect of this procedure consists in the very different severity of TNBS colitis and accompanying profile of inflammatory cytokines in the two lineages. In fact, TNBS colitis that was more severe in *Nod2*+/+ mice reared separately, is now less severe than the one observed in *Nod2−/−*, while the opposite is true for *Nod2−/−* mice.

Comparing the abundance of phyla between these four groups of mice, some significant differences emerged (Fig. 6 and Supplementary Table S1). First, comparison between the pair of less severe colitis (observed in *Nod2*−/− reared separately and in *Nod2*+/+ mice cohoused), showed a certain balance in the Bacteroidetes/Firmicutes ratio, suggesting that in *Nod2*+/+ cohoused mice the acquisition of *Nod2−/−* microbiota expands the Firmicutes phyla (Supplementary Table S1), under the pressure of the inflammatory agent TNBS. It is interesting to note that Firmicutes were indeed more represented in *Nod2−/−* mice at 4 weeks of age (Fig. 2D and Supplementary Table S1). Similarly, acquisition of *Nod2−/−* microbiota by *Nod2*+/+ mice was associated, as in *Nod2*+/+ mice reared separately, with expansion of Proteobacteria and reduction of Firmicutes and Bacteroidetes (Fig. 6 and Supplementary Table S1), that, altogether, represented about the 95–98% of bacteria at 4 weeks and 8 weeks of age in both housing conditions. As known^30^, unregulated intestinal inflammation results in an environment favouring bacterial expansion of Proteobacteria (44% in *Nod2−/−* cohoused and 20% in *Nod2*+/+ mice reared separately) and corresponding reduction in Firmicutes. This was corroborated by a dominance of *Escherichia/Shigella* and *Enterococcus* genera observed in severe colitis (*Nod2*+/+ mice reared separately and co-housed *Nod2−/−* mice), and a reduction of *Barnesiella* (Fig. 5D, E; Supplementary Table S2), involved in protection from experimental colitis^46^.

Less severe colitis (*Nod2*+/+ cohoused mice and in *Nod2−/−* mice reared separately) was associated with no expansion of Proteobacteria (under 1–2%; Supplementary Table S1), and enrichment of *Clostridium cluster IV*, that include *F*. *prausnitzii*, a known bacteria with anti-inflammatory properties and *Odoribacter*, a known SCFA-producer that have trophic function on epithelial cells^47^, in the respective groups (Fig. 5D, E).

Regarding functional contribution of microbial communities that could be associated with severity of colitis, LEfSe analysis performed on PICRUSt prediction confirmed an enrichment in functions related to membrane and intracellular structural molecules, especially LPS biosynthesis, cell motility (Flagellar assembly, Bacterial chemotaxis and Bacterial motility proteins) in *Nod2−/−* cohoused mice (Supplementary Fig. S4), and an enrichment in Arachidonic acid metabolism in microbiota of *Nod2*+/+ mice reared separately (Supplementary Fig. S5). Both LPS, from Gram-negative bacteria, and metabolites of arachidonic acids have immunostimulatory and endotoxic properties which mediate inflammatory reactions^48^.

The severe condition of colitis presents in *Nod2−/−* mice co-housed with the *Nod2*+/+ has characteristics similar to microbial dysbiosis presents in CD patients^47^. Both conditions has been characterized by a higher abundance of Proteobacteria, a reduction of Bacteroidetes and a lower abundance of Firmicutes, particularly within the *Clostridium XIVa* and *IV* clusters^23,49^. Studies of correlation between relative abundance of bacterial taxa and number of minor alleles at known IBD risk loci, including fine mapping of multiple risk alleles in NOD2 gene exon, identified a significant association between NOD2 risk allele count and increased relative abundance of *Enterobacteriaceae*^50^. Thus, genetic influence indeed contributes to the inflammation-mediated dysbiosis.

**Figure 7:**
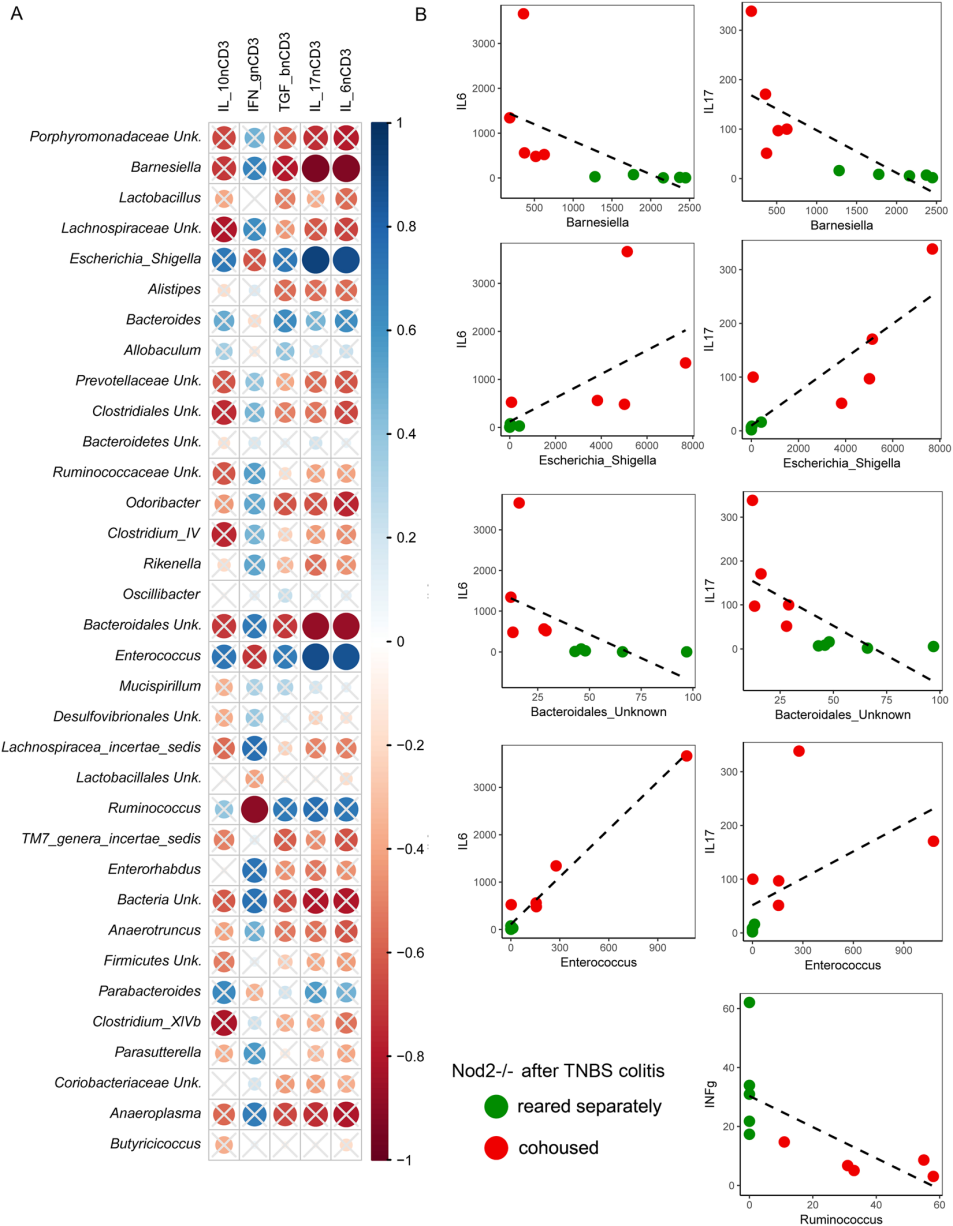
Correlation analyses among bacterial taxa abundance and mucosal cytokines in *Nod2*−/− mice after TNBS-induced colitis. (**A**) Spearman’s r correlation analysis among bacterial relative abundances (taxa with relative abundance <0.1%) and mucosal cytokines expression measured by RT-qPCR (IL-10, INF-γ, TGF-β, IL-17 and IL-6 normalized using the HK gene CD3). Solid circles represent the degree of correlation among the variables taken into account. Crossed circles indicate non-significant correlations; statistically significant results are indicated with p < 0.05. (**B**) Correlation analysis among bacterial taxa and mucosal cytokines according to mild and severe colitis in *Nod2*−/− mice in different housing conditions.

The above observations suggest that genetic differences might induce differences in the “core” microbiota profile. These differences influence the generation of regulatory T cells, particularly CD4+ LAP+ Foxp3− cells that are responsible for the response to inflammatory stimuli. As a consequence of different severity of inflammation, the microbiota of inflamed mice showed different prevalence of bacterial taxa. This hypothesis was corroborated by the correlation we observed with different tissue content of cytokines. In fact, *Barnesiella* and *Bacteroidales_unknown* were inversely correlated with IL-6 and IL-17, and *Ruminococcus* with IFN-γ, while *Escherichia/Shigella* and *Enterococcus* were directly correlated with IL-6 and IL-17 (Fig. 7).

## Conclusions

Taken together the data lead to the following major conclusions: (i) *Nod2* deficiency is characterized by increased immune-regulation associated with a highly diverse microbial profiles that favours expansion of regulatory cells, particularly CD4+ LAP+ Foxp3− cells, preventing the onset of intestinal inflammation in response to mild (TNBS) external stimuli; (ii) environmental factors (cohousing) intervening between the age of weaning and young adulthood (4–8 weeks of age), a period characterized by microbiota instability, favours the sharing of microbial species. *Nod2−/−* endow potentially useful microbes beneficial for *Nod2*+/+ when colitis occurs, providing them with more “volatile” microbial families and genera, most likely linked to the relative short time of colonization. On the other hand, *Nod2*+/+ can expose *Nod2−/−* to microbial species that these cannot normally control, and that can be potentially harmful during colitis.

Thus, at least in certain circumstances, co-housing is able to alter the genetic-induced microbiota composition, through reduction of microbial richness and expansion of Proteobacteria to the detriment of the main phyla Bacteroidetes and Firmicutes, potentially contributing to the onset of a severe inflammation in *Nod2−/−*. This notion might be of relevance in the therapeutic attempt to re-establish eubiosis in CD patients with NOD2 polymorphisms.

## Methods

### Animals, housing conditions and sampling of feces and tissues

*Mice*. *Nod2*+/+ and *Nod2−/−* mice on C57BL/6 background were kindly donated by Peter J Murray, Department of Infectious Diseases and the Department of Immunology, St Jude Children’s Research Hospital, Memphis, Tennessee, USA. At the Istituto Superiore di Sanità (Rome, Italy), *Nod2−/−* mice and control *Nod2*+/+ mice had been re-derived by embryo transfer into littermate foster mothers raised in the same SPF animal facility and in the same cage. *Nod2−/−* and *Nod2*+/+ mice were maintained in different cages and trio breeding groups (defined as one adult male and two adult females) have been used to obtain better performance in the breeding. *Nod2−/−* and *Nod2*+/+ mice were maintained in SPF conditions in the animal facility at the Istituto Superiore di Sanità. All mice were housed with free access to food and water. Pathogen-free conditions were monitored every 6 months in accordance with the full set of FELASA high standards recommendations. All animal studies were approved by the Animal Care and Use Committee of the Istituto Superiore di Sanità. All procedures were performed in accordance with the Guide for the Care and Use of Laboratory Animals and were approved by the Italian Ministry of Health (Reference: n. 4 42 /2015-PR).

A total of 50 mice were included in the experimental design, as following: at time of weaning (4 weeks of age), *Nod2*+/+ (n = 25) and *Nod2−/−* (n = 25) males mice have been separated from their mothers and distributed in new cages so that each cage contained mice born to different mothers. A total of 5 mice/group were scarified for cytokine mRNA tissue content and proportion of LP CD4+ LAP+ Foxp3− regulatory cells. Groups of *Nod2−/−* (n = 10) and *Nod2*+/+ (n = 10) mice were reared separately, while other groups were co-housed in the same cage for 4 weeks (n = 10 *Nod2−/−* mice and n = 10 *Nod2*+/+ mice).

At 8 weeks of age, a total of 5 mice/group were scarified for cytokine mRNA tissue content and proportion of LP CD4+ LAP+ Foxp3− regulatory cells. The remaining mice (n = 10 *Nod2−/−* and n = 10 *Nod2*+/+) reared separately or co-housed, colitis was induced by administration of 2,4,6-trinitrobenzenesulfonic acid (TNBS) (3.5 mg; Sigma-Aldrich, Saint Louis, USA) delivered in 150 µl of 50% ethanol per rectum as previously described^12^.

#### Faeces samples collection

Faeces were collected at different time points: (i) at 4 weeks of age (at weaning, before the housing conditions were established; n = 5 faecal samples from *Nod2*+/+ and n = 5 from *Nod2−/−* mice); (ii) at 8 weeks of age (before TNBS-colitis induction; n = 10 faecal samples from *Nod2*+/+ mice and n = 10 from *Nod2−/−* mice, randomized to cohousing and no-cohousing conditions), and (iii) at day 3 after TNBS colitis induction (n = 10 faecal samples from *Nod2*+/+ mice and n = 10 faecal samples from *Nod2−/−* mice equally distributed in different housing conditions). A total of 50 faecal samples were collected immediately after defaecation and stored at −80 °C until analysed for microbiota composition. Some mice belonging to the different groups were sacrificed at 4, 8 weeks of age and at day 3 after TNBS-colitis induction for evaluation of tissue cytokines and at 4 and 8 weeks of age for evaluation of LP regulatory T cells.

#### Tissue collection

Groups of 5 mice from different housing conditions were sacrificed at 4 and 8 weeks of age. The remaining mice (n = 10 *Nod2*+/+ mice and n = 10 from *Nod2−/−* mice in different housing conditions) were sacrificed at day 3 after TNBS-colitis induction. Colons were collected for the evaluation of the frequency of regulatory cells from Lamina Propria Mononuclear Cells (LPMC) as previously described^12^ and for the assessment of gene expression of cytokine by qPCR (Fig. 1 and Table 1). In detail, tissue samples for the assessment of gene expression of cytokine by qPCR were preliminary obtained, in each mouse, after cecum removal, from the mid colon. The remaining colonic segments (proximal and distal colon) were pooled and further processed for LPMC isolation.

### RNA extraction, reverse transcription and qPCR

Tissue sample were collected from each mouse in RNAlater (Ambion - Thermo Fisher Scientific, Europe), stored overnight at 4 °C and then stored at −80 °C until RNA extraction. Total RNA extraction was performed by RNeasy Mini Kit Plus (Qiagen) according with manufacturer’s instructions. RNA quality was assessed by gel electrophoresis and spectrophotometry measuring OD 260/280. 1μg of RNA/sample was retrotranscribed into complementary DNA (cDNA) using TaqMan® Reverse Transcription Reagents (ThermoFisher Scientific). Cytokine of interest was then amplified by qPCR using Syber Green chemistry (Fast SYBR Green Master mix, ThermoFisher Scientific). Each sample was processed with technical triplicates. Reactions were run in a ViiA™ 7 Real-Time PCR System (Applied Biosystems). All primer sets were designed to span an intron and utilized for real-time PCR (Supplementary Table S3). Cytokine RNA expression was calculated relative to the housekeeping HPRT gene on the basis of the ΔΔCt algorithm^51^ and expressed as Arbitrary Units (AU).

### Bacterial DNA extraction

For each group of mice, the pellets were collected in sterile conditions and stored at −80 °C until extraction of nucleic acids. The bacterial genomic DNA extraction was carried out with FastDNA SpinKit for feces (MP Biomedicals, Santa Ana, CA, USA) following the manufacture’s instructions. DNA quality was assessed by gel electrophoresis and spectrophotometry, measuring OD 260/280.

### Pyrosequencing and data analysis

The 16S rRNA gene was amplified using the special fusion primer set specific for V3−V5 hypervariable regions, and the FastStart High Fidelity PCR system (Roche Life Science, Milano, Italy), as previously described^52^. The 454 pyrosequencing was carried out on the GS FLX + system (Roche) using the XL + chemistry following the manufacture’s recommendations. We obtained a total of 1,453,737 16S rDNA reads, with a mean of 29,074.74 sequences per sample. Average sequence lengths were 586 nt (±SD 32). Raw 454 files were demultiplexed using Roche’s.sff file software. Reads were pre-processed using the MICCA pipeline (version 1.5, http://compmetagen.github.io/micca/)^53^. Forward and reverse primer trimming and quality filtering were performed using micca-preproc truncating reads shorter than 300 nt (quality threshold = 18). *Denovo* sequence clustering, chimera filtering and taxonomy assignment were performed by micca-otu-denovo. Operational Taxonomic Units (OTUs) were assigned by clustering the sequences with a threshold of 97% pair-wise identity. The representative sequences were classified using the RDP classifier version 2.7 against RDP 11 database (update 5) of 16S rRNA. Template-guided multiple sequence alignment was performed using PyNAST (version 0.1) against the multiple alignment of the Greengenes 16S rRNA gene database (release 13_05) and filtering at 97% similarity.

A total of 943 Operational Taxonomic Units (OTUs) were assigned by clustering the sequences with a threshold of 97% pair-wise identity.

Alpha diversity was estimated by Observed OTUs and Shannon entropy. Rarefaction curves were calculated by number of observed OTUs for different values of the rarefaction depth for each sequenced sample by using PAST3 tool, in order to evaluate if the sequences were sufficient to cover the biodiversity of microbial communities (Supplementary Fig. S6). Sampling heterogeneity was reduced by rarefaction, obtaining 10‘141 sequences per sample (Supplementary Datafile 1).

Beta diversity was performed to evaluate differences in overall bacterial communities by Principal Coordinates Analysis (PCoA) and Non-metric Multidimensional Scaling (NMDS), based on Bray-Curtis dissimilarities, using the phyloseq package of the R software suite^54^. The significance of between-groups differentiation on Bray-Curtis dissimilarity was assessed by PERMANOVA using the adonis() function of the R package vegan with 999 permutations.

To infer the functional contribution of microbial communities (revealing functional classes; KEGG categories) on 16S rDNA sequencing data set, we applied Phylogenetic Investigation of Communities by Reconstruction of Unobserved States- PICRUSt^29^, a computational approach useful to infer the functional contribution of microbiome based on 16S rRNA dataset. We obtained the final output from metagenome prediction as an annotated table of predicted gene family counts for each sample, where the encoded function of each gene family be orthologous groups or other identifiers such as KEGG orthologs (KOs).

### Statistical analyses

Statistical differences in mice body weight and in cytokine RNA expression data were evaluated using parametric and non-parametric statistic as appropriated through GraphPad Prism software (GraphPad Software, San Diego, CA). A p-value <0.05 was considered statistically significant.

Metagenomic biomarker discovery and related statistical significance were assessed by using the linear discriminant analysis (LDA) effect size (LEfSe) method^55^, based on the bacterial relative abundances. In LEfSe, Kruskal–Wallis rank-sum test is used to identify significantly different taxa abundances among groups of mice considering different genotype and stabulation condition (cohousing or reared separately), and LDA to estimate the size effect of each feature. An alpha significance level of 0.05, either for the factorial Kruskal-Wallis test among classes or for the pairwise Wilcoxon test between subclasses, was used. A size-effect threshold of 2.0 on the logarithmic LDA score was applied for discriminative microbial biomarkers. LEfSe was also performed on PICRUSt data to discover bacterial functional biomarkers among groups of mice based on different genotype and stabulation conditions.

Spearman’s r correlation analysis was performed to correlate bacterial relative abundances (with relative abundance > 0.1%) and cytokines expression (RT-qPCR on IL-6, IL-17, IFN-γ, IL-10 and TGF-β normalized using the HK gene CD3) under different conditions (stabulation, genetic background, colitis). Spearman’s correlation tests were computed using the psych R package^56^ and p-values were corrected for multiple comparison controlling the false discovery rate^57^.

## Electronic supplementary material

Supplementary Information

Supplementary Table S2

Supplementary datafile1

## Data Availability

Raw 454 files are available at the European Nucleotide Archive under the accession study PRJEB22726.
