# Supplementary material for: Nod2 Deficiency in mice is Associated with Microbiota Variation Favouring the Expansion of mucosal CD4+ LAP+ Regulatory Cells
Source: Sci Rep. 2018 Sep 24;8:14241. doi: 10.1038/s41598-018-32583-z (PMC6155205; doi:10.1038/s41598-018-32583-z)
Supplement: Supplementary file 1 — Supplementary Information [file 41598_2018_32583_MOESM1_ESM.pdf]

Supplementary Information

Nod2 Deficiency in mice is Associated with Microbiota Variation Favouring the Expansion of mucosal CD4+LAP+ Regulatory Cells

A. Butera[1]*, M. Di Paola[2,3]*, L. Pavarini[4], F. Strati[5], M. Pindo[4], M. Sanchez[6], D. Cavalieri[3], M. Boirivant[1§], C. De Filippo[7§]

1. Pharmacological Research and Experimental Therapy Section, National Center for Drug Research and Evaluation, Istituto Superiore di Sanità, Rome, Italy

2. Department of Neuroscience, Psychology, Drug Research and Child Health (NEUROFARBA), University of Florence, Meyer Children Hospital, Florence, Italy;

3. Department of Biology, University of Florence, Sesto Fiorentino, Florence, Italy

4. Research and Innovation Centre, Fondazione E. Mach, S. Michele all’Adige, Trento, Italy

5. Institute for Research in Biomedicine (IRB), Università della Svizzera italiana, Bellinzona, Switzerland

6. Cytometry Unit - Core Facilities, Istituto Superiore di Sanità, Rome, Italy

7. Institute of Biology and Agrarian Biotechnology (IBBA), National Research Council (CNR), Pisa, Italy.

*equally contributed to the study

§ CDF and MB share senior authorship

Supplementary Table

Supplementary Table S1. Proportion of the major phyla (Bacteroidetes, Firmicutes and Proteobacteria) in *Nod2-/-* and *Nod2+/+* mice in different housing condition at 4 weeks, 8 weeks and after TNBS colitis.

	4 weeks		
	phylum	abundance	B/F ratio
WT	Bacteroidetes	72.6%	2.86
	Firmicutes	25.4%	
	Proteobacteria	0.4%	
NOD2-/-	Bacteroidetes	48.9%	1.05
	Firmicutes	46.6%	
	Proteobacteria	1.0%	

		8 weeks			colitis			
		phylum	abundance	B/F ratio	phylum	abundance	B/F ratio	colitis
cohousing	WT	Bacteroidetes	54%	1.26	Bacteroidetes	50%	1.04	mild
		Firmicutes	43%		Firmicutes	48%		
		Proteobacteria	0.7%		Proteobacteria	1%		
	NOD2-/-	Bacteroidetes	68%	2.19	Bacteroidetes	31%	1.29	severe
		Firmicutes	31%		Firmicutes	24%		
		Proteobacteria	0.6%		Proteobacteria	44%		
reared separately	NOD2-/-	Bacteroidetes	57%	1.43	Bacteroidetes	66%	2.13	mild
		Firmicutes	40%		Firmicutes	31%		
		Proteobacteria	1%		Proteobacteria	2%		
	WT	Bacteroidetes	71%	2.63	Bacteroidetes	49%	1.63	severe
		Firmicutes	27%		Firmicutes	30%		
		Proteobacteria	0.7%		Proteobacteria	20%		

Supplementary Table S2 (datafile).
Summary of the relative abundance percentages of the taxa (at Phylum, Family and Genus level) in microbiota of *Nod2-/-* and *Nod2+/+* mice in the different housing conditions. Percentages related to each Phylum relative abundance (grey) correspond to sum of the relative abundance of families belonging to the respective phylum. Percentages related to each family relative abundance (light grey) correspond to sum of the relative abundance of genera belonging to the respective family. In red, we indicated the statistically significant abundances, as resulted by LEfSe analyses.

Supplementary Table S3. Primers used in RT-PCR for cytokine RNA expression.

Primer	Forward	Reverse
HPRT	5’-CTGGTGAAAAGGACCTCTCG-3’	5’-TGAAGTACTCATTATAGTCAAGGGCA-3’
IL10	5’-GGTTGCCAAGCCTTATCGGA-3’	5’-ACCTGCTCCACTGCCTTGCT-3’
TGF-β	5’-ACCGCAACAACGCCATCTAT-3’	5’-GCAACAATTCCTGGCGTTAC-3’
IL-23p19	5’-CAGCGGGACATATGAATCTA-3’	5’-CCTTGAGTCCTTGTGGGTCA-3'
IL-6	5’-GCAACAATTCCTGGCGTTAC-3’	5’-GAAGTAGGGAAGGCCGTGG-3’
IL-17A	5’-TCAGACTACCTCAACCGTTC-3’	5’-TTCAGGACCAGGATCTCTTG-3’

Datafile_1. Microbiota profiles at different taxonomic levels. Rarefied OTU tables at (A) phylum, (B) family and (C) genus level obtained for each sequenced sample were reported.

Supplementary Figures

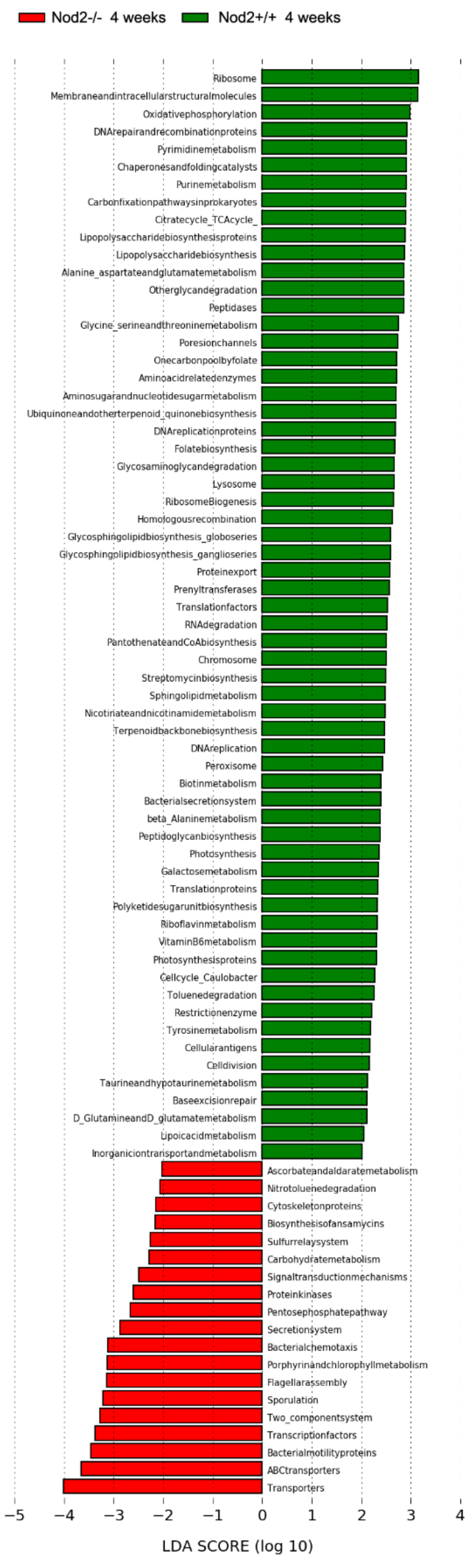

Supplementary Figure S1. Metabolic function prediction associated to the microbiota profiles in Nod2-/- and Nod2+/+ mice at 4 weeks of age. LEfSe analysis, performed on metabolic functions inferred by PICRUSt, shows statistically significant enrichment of KEGG categories in Nod2-/- and Nod2+/+ mice (Alpha value=0.05 for the factorial Kruskal–Wallis test among classes; the threshold for the logarithmic LDA score=2.0).

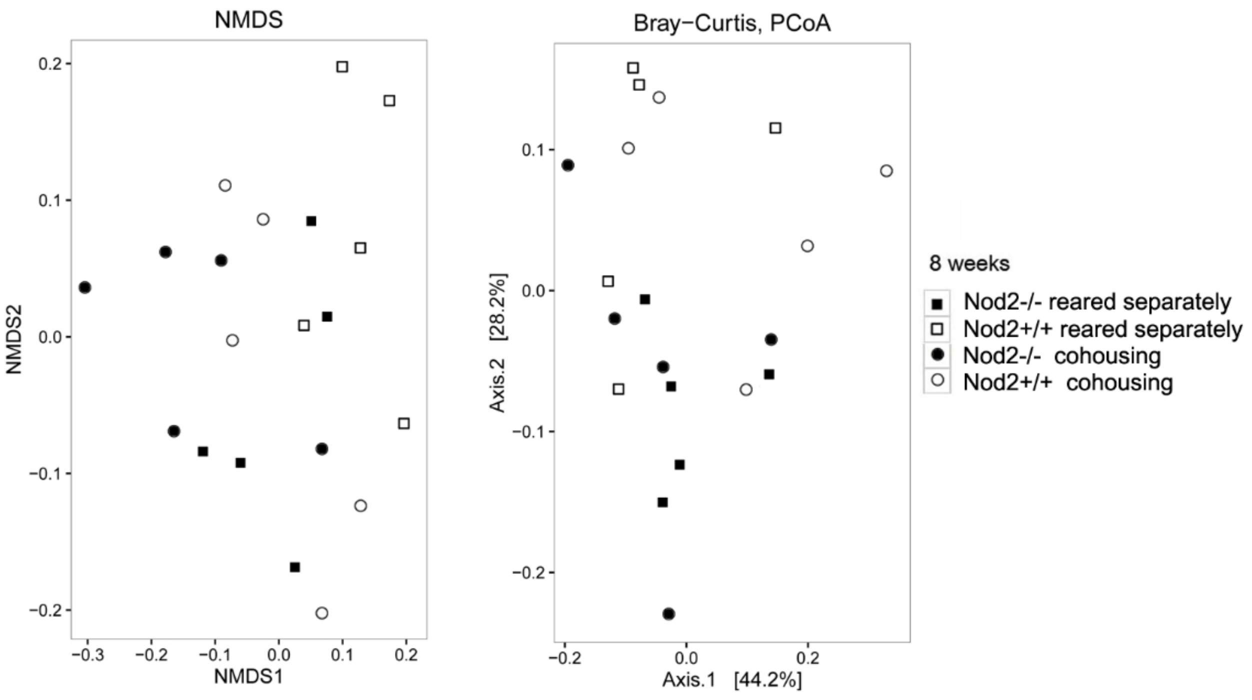

Supplementary Figure S2. Beta diversity measure. Comparison of microbial variability among faecal samples of Nod2-/- and Nod2+/+ mice at 8 weeks of age in different housing conditions. PCoA obtained by NMDS and Bray-Curtis distances (statistical significance by PERMANOVA, p=0.001).

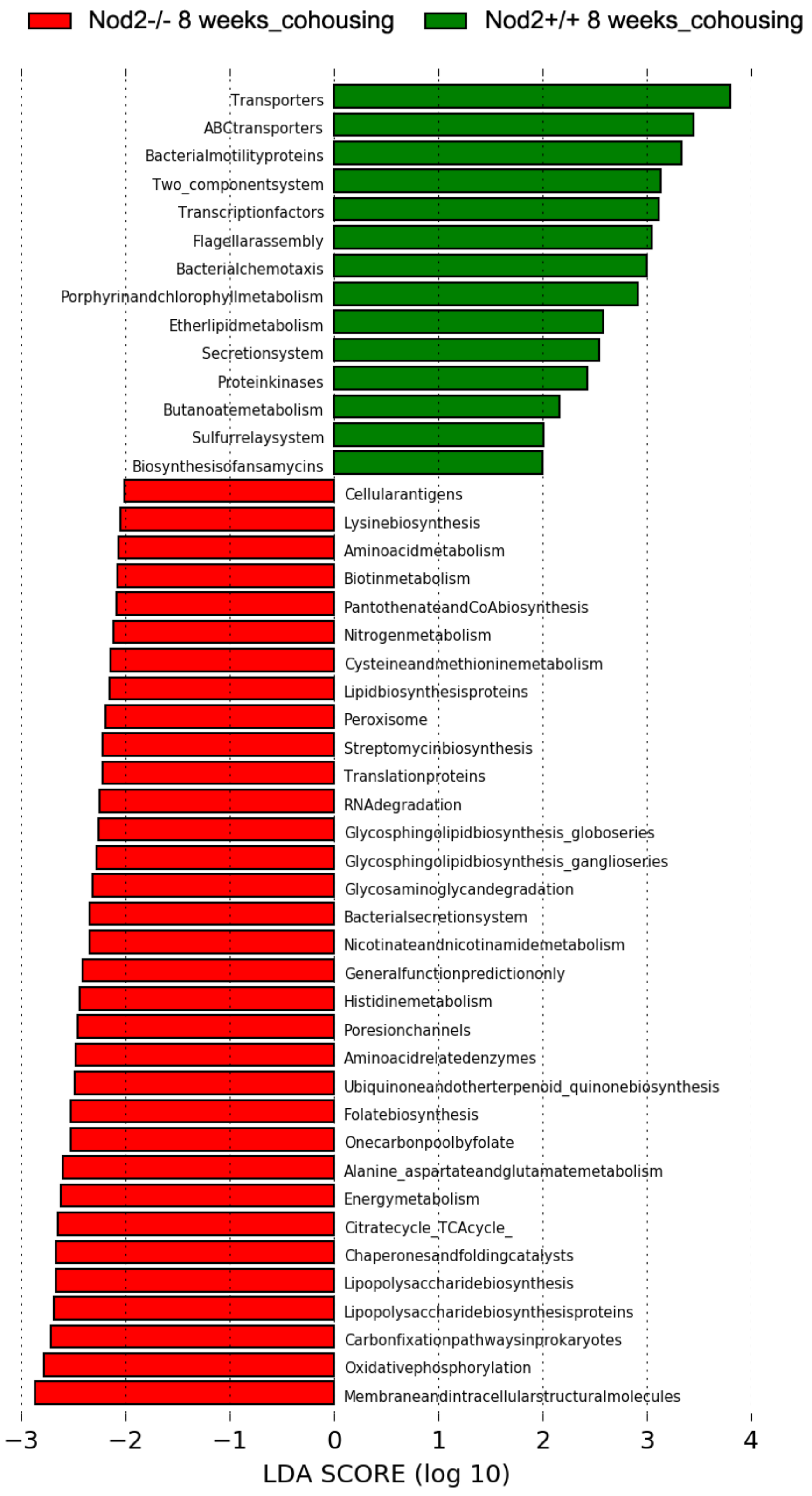

Supplementary Figure S3. Metabolic function prediction associated to the microbiota profiles in Nod2-/- and Nod2+/+ mice at 8 weeks of age in cohousing condition. LEfSe analysis, performed on metabolic functions inferred by PICRUSt, shows statistically significant enrichment of KEGG categories (Alpha value=0.05 for the factorial Kruskal–Wallis test among classes; the threshold for the logarithmic LDA score=2.0).

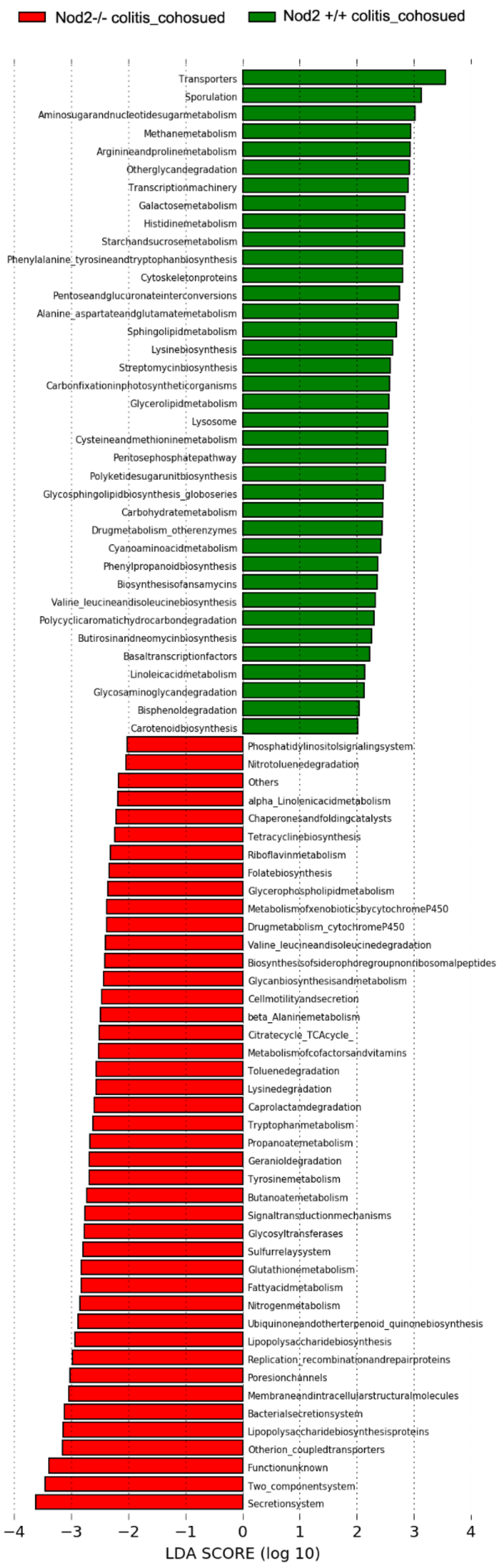

Supplementary Fig. S4 Metabolic function prediction associated to the microbiota profiles in Nod2-/- and Nod2+/+ mice in cohousing condition, after TNBS-colitis. LEfSe analysis, performed on metabolic functions inferred by PICRUSt, shows statistically significant enrichment of KEGG categories (Alpha value=0.05 for the factorial Kruskal–Wallis test among classes; the threshold for the logarithmic LDA score=2.0).

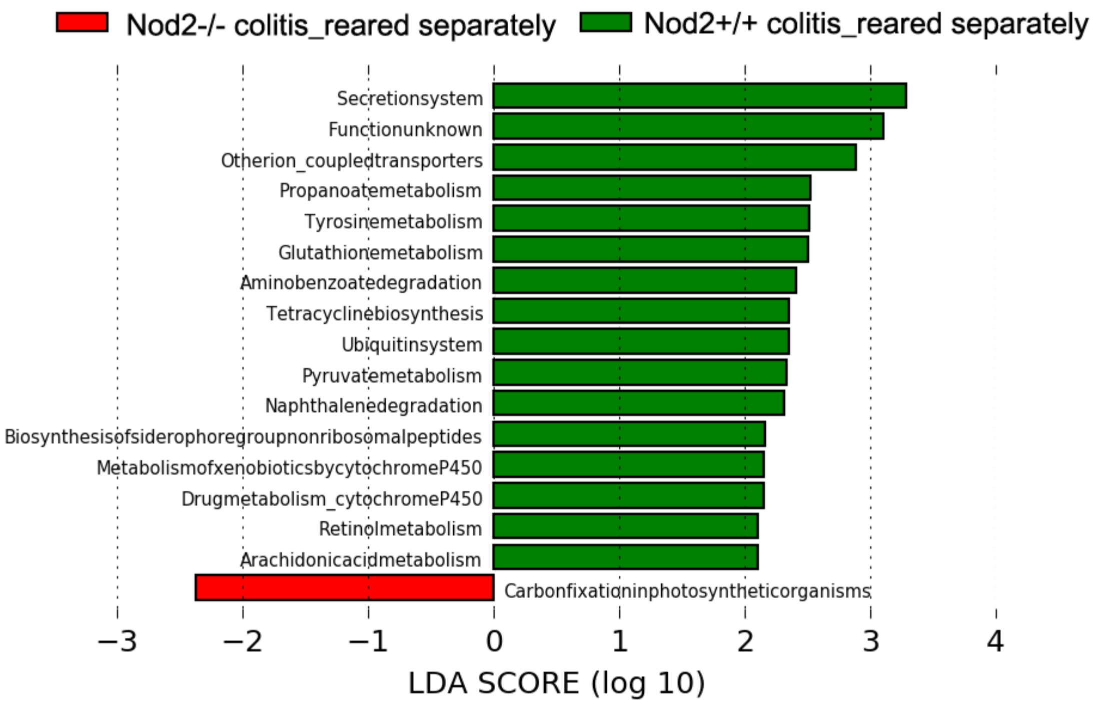

Supplementary Fig. S5 Metabolic function prediction associated to the microbiota profiles in Nod2-/- and Nod2+/+ mice reared separately, after TNBS-colitis. LEfSe analysis, performed on metabolic functions inferred by PICRUSt, shows statistically significant enrichment of KEGG categories (Alpha value=0.05 for the factorial Kruskal–Wallis test among classes; the threshold for the logarithmic LDA score=2.0).

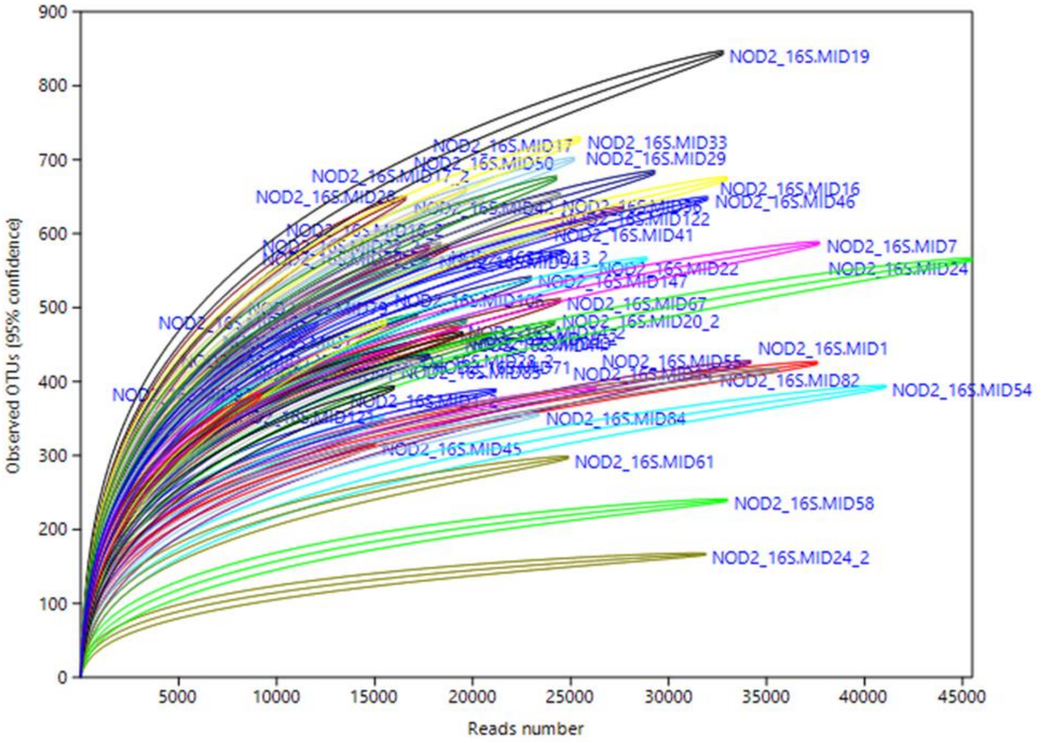

Supplementary Fig. S6 Rarefaction curves based on number of observed OTUs for each sequenced sample, obtained by PAST3 tool.
